# Perinatal Bisphenol A Exposure and Adult Glucose Homeostasis: Identifying Critical Windows of Exposure

**DOI:** 10.1371/journal.pone.0064143

**Published:** 2013-05-10

**Authors:** Jingli Liu, Pan Yu, Wenyi Qian, Yan Li, Jingjing Zhao, Fei Huan, Jun Wang, Hang Xiao

**Affiliations:** Department of Toxicology, School of Public Health, Nanjing Medical University, Nanjing, China; Universidad Miguel Hernández de Elche, Spain

## Abstract

Bisphenol A (BPA) is a widespread endocrine-disrupting chemical used as the building block for polycarbonate plastics. Epidemiological evidence has correlated BPA exposure with higher risk of heart disease and type 2 diabetes. However, it remains unknown whether there are critical windows of susceptibility to BPA exposure on the development of dysglycemia. This study was an attempt to investigate the critical windows and the long-term consequences of perinatal exposure to BPA on glucose homeostasis. Pregnant mice were given either vehicle or BPA (100 µg/kg/day) at different time of perinatal stage: 1) on days 1–6 of pregnancy (P1–P6, preimplantation exposure); 2) from day 6 of pregnancy until postnatal day (PND) 0 (P6–PND0, fetal exposure); 3) from lactation until weaning (PND0–PND21, neonatal exposure); and 4) from day 6 of gestation until weaning (P6–PND21, fetal and neonatal exposure). At 3, 6 and 8 months of age, offspring in each group were challenged with glucose and insulin tolerance tests. Then islet morphometry and β-cell function were measured. The glucose homeostasis was impaired in P6-PND0 mice from 3 to 6 months of age, and this continued to 8 months in males, but not females. While in PND0-PND21 and P6-PND21 BPA-treated groups, only the 3-month-old male offspring developed glucose intolerance. Moreover, at the age of 3 months, perinatal exposure to BPA resulted in the increase of β-cell mass mainly due to the coordinate changes in cell replication, neogenesis, and apoptosis. The alterations of insulin secretion and insulin sensitivity, rather than β-cell mass, were consistent with the development of glucose intolerance. Our findings suggest that BPA may contribute to metabolic disorders relevant to glucose homeostasis and the effects of BPA were dose, sex, and time-dependent. Fetal development stage may be the critical window of susceptibility to BPA exposure.

## Introduction

Diabetes represents a growing public health concern in both industrialized and developing countries. This rapid outbreak cannot only be explained by genetic predisposition, but also be related to nutrition, changes in physical activity, or environmental modifications and so on. During the last decade, accumulating evidences show that adverse environmental factors exposed during early development play an important role in determining the risk of developing chronic diseases in adulthood. Initially, the concept of “fetal origin of adult diseases” suggested that a mismatch between fetal expectation of the postnatal environment and actual postnatal environment could contribute to adult disease risk [1]. For example, maternal protein deficiency or undernutrition, predisposes offspring to obesity and insulin resistance at adulthood [2], [3]. Besides, Voluntary or passive exposure to chemical pollutants is more relevant to diabetes in our modern life. Recently, environmental estrogens such as bisphenol A (BPA) have become public health concerns because of their deleterious effects on energy balance and glucose homeostasis in animal models [4], [5].

BPA (2,2-bis-(4-hydroxyphenyl) propane) is a base compound in the manufacture of polycarbonate plastic and resins used as lining for metal cans as well as other widely used plastics such as polyvinyl chloride and polyethylene terephthalate [6]. Widespread and continuous human exposure to BPA has been reported. It was found in the urine of 95% of US citizens [7] and its concentration in human serum ranges from 0.3 to 4 ng/ml [8]. In addition, it has been detected in amniotic fluid, neonatal blood, placenta, cord blood and human breast milk, demonstrating the potential of this compound to pass from mother to fetus [9]. Therefore, maternal BPA exposure results in both fetal and neonatal exposure.

Recently, epidemiological studies have indicated that BPA has been associated with type 2 diabetes and cardiovascular diseases [10]. Animal studies also mount in support of the concept that BPA perinatal exposure induces metabolic disturbances. However, the effects of BPA are still in debate. It is reported that male mice offspring exposed to 10 µg/kg/d BPA during days 9–16 of gestation show altered blood parameters, impaired glucose tolerance at 6 months old [11]. Perinatal exposure (P6-PND21) to 1 mg/L BPA via drinking is also observed to alter early adipogenesis in the rat at weaning [12]. However, perinatal exposure to BPA at approximately 0.25 µg/kg/d via the diet has not been shown to impair glucose tolerance or increase body weight at adult, even when tested on a high-fat diet, but mice are longer than controls at 4 weeks old [13]. Some other studies also show different effects of BPA exposure on body weight [5], [14], [15], [16]. These differences might be attributed to the varied species, dose, route of administration, and the time of exposure.

In humans, BPA exposure routinely occurs via residues contained in food, beverages or dental materials. Although ingestion is considered the major route of exposure, it is possible that additional routes (inhalation and contact) could also be the significant source of exposure [17]. There is remarkable leaching of BPA from children’s books, thermal (carbonless) receipts, CDs et al. Recently, some pharmacokinetic experiments show that BPA exposure may be much higher than initially thought [8], [18], [19] and skin absorption route may be put forward to explain this inconsistency [20]. Therefore, subcutaneous injection was chosen for this study to mimic the important nonfood sources of BPA exposure in humans and wildlife. In addition, the dose of 100 µg/kg BW BPA used in this study was below the current lowest observed effect level (LOAEL) of 50 mg/kg/day, established by the U.S. Environmental Protection Agency (EPA) [8]. Despite it is the fact that this dose cannot be considered exactly environmentally relevant, it is considered low dose for in vivo studies [21].

Whether the different developmental stages of BPA exposure can result in dissimilar impact on glucose homeostasis in the offspring have not yet been clarified. Therefore, this study was designed to delineate critical windows of BPA exposure on the development of the pancreas and glucose intolerance in the offspring. Meanwhile, the long-term consequences of perinatal exposure to BPA on glucose homeostasis were observed as well.

## Materials and Methods

### Animals and Treatment

All experiments involving animals and tissue samples were conducted in accordance with the guide for the Care and Use of Laboratory Animals of the National Institutes of Health (NIH) (USA), and all procedures were approved by the Institutional Animal Care and Use Committee (IACUC) of Nanjing Medical University (China) (Permit Number: 20110521).

Male and female C57BL6 mice (F0), 8 weeks old, were purchased from Experimental Animal Center of Nanjing Medical University (Nanjing, China). The mice were housed throughout the experiments under specific-pathogen-free (SPF) conditions, with controlled illumination (12 h light: 12 h dark cycles), humidity (30–50%), and temperature (18–22°C). Both glass water bottles and BPA free polypropylene cages were used in this study. And the diet did not contain alfalfa or soybean meal, eliminating the influence of phytoestrogens. After more than one-week acclimation period, the mice were paired (one male: one female). Observation for mating during the preceding night was made by inspecting females for the presence of a vaginal plug from 8∶00 am to 10∶00 am in the next morning.

The day that vaginal plug was observed in mated females was declared d1 pregnancy (P1), and the day of parturition was PND0. Pregnant mice were housed individually and randomly assigned (n>10 per group) to receive corn oil (vehicle) or BPA (100 µg/kg BW per day; Sigma-Aldrich) via s.c. injection daily: 1) on days 1–6 of pregnancy (P1–P6, preimplantation exposure); 2) from day 6 of pregnancy until parturition (P6-PND0, fetal exposure); 3) from lactation until weaning (PND0-PND21, neonatal exposure); and 4) from day 6 of gestation until weaning (P6-PND21, fetal and neonatal exposure). The control animals received isovolumic (about 50 µl) vehicle during P1-PND21, as this volume of corn oil did not affect glucose metabolism, which was verified by our previous experiments performed in mice. At PND0, litters were culled to six, assuring uniformity of litter size between treated and control litters.

### Measurement of Pregnancy Outcomes

Pregnant mice from control and BPA-treated groups were visually monitored daily for the anticipated duration of pregnancy (20–21 days). Full-term pregnancies resulting in births of pups (F1) were marked as successful. Females that did not reach pregnancy at term, and for whom no signs of birth were observed, were marked as nonproductive. Pups were weighed on postnatal days 0, 21, and then weighed weekly till 8 months.

### Glucose and Insulin Tolerance Tests

Glucose homeostasis was investigated in F1 mice of control and BPA-treated groups at the age of 3 months, 6 months and 8 months using intraperitoneal glucose tolerance tests (IPGTT). Mice were fasted overnight for 14–16 h, and then injected intraperitoneally with glucose at 2 g/kg body weight. Blood samples were collected from the angular vein at 0, 15, 30, 60 and 120 min. Blood glucose was measured in each sample using an automatic glucometer (Roche). The area under the curve (AUC) was calculated as an index of glucose tolerance.

The plasma was then collected for insulin detection by enzyme linked immunosorbent assay (Millipore). The insulinogenic index (ΔI_0–30_/ΔG_0–30_), an index of insulin secretion function in response to glucose in vivo, was calculated as change in insulin from 0 to 30 minutes divided by change in glucose over the same time. The formula is as follows: ΔI_0–30_/ΔG_0–30_ = (I_30_–I_0_)/(G_30_–G_0_) [22].

For the intraperitoneal insulin tolerance tests (IPITT), fed animals were injected i.p. with 1I U/kg body weight soluble insulin from 13∶00 pm to 14∶00 pm. Blood glucose was measured at various time points (0, 15, 30, 45 and 60 min).

### Glucose-stimulated Insulin Secretion (GSIS) from Isolated Islets

Pancreatic islets of Langerhans were isolated by collagenase (Sigma-Aldrich) digestion as previously described [23] and cultured in groups in RPMI 1640 without phenol-red, containing 11 mM glucose (Gibco) at 37°C in a humidified atmosphere of 95% O_2_ and 5% CO_2_. The medium was supplemented with 10% fetal bovine serum (Hyclone), 2 mM L-glutamine, 200 U/ml penicillin and 0.2 mg/ml streptomycin.

After the overnight incubation, groups of 20 islets of similar size were incubated in Krebs-Ringer bicarbonate (KRB) buffer containing 0, 3.3 or 16.7 mmol/L glucose for 1 h. Insulin secreted in the media was assayed by RIA. Protein concentration was measured by the Bradford dye method.

### Islet Morphometric Analysis

To assess whether BPA exposure alters postnatal development of pancreas, pancreatic tissue was obtained from 3-month-old and 8-month-old mice for immunohistochemistry. All mice were sacrificed by CO_2_ inhalation. The pancreas was weighed and then fixed in 10% (v/v) neutral formalin at room temperature for 24 h, and embedded in paraffin. Detection of β cells by rabbit anti-mouse insulin antibody (1∶200 dilution; CST) was performed on 5 µm serial sections, separated by an average of 30 µm. Five sections per animal and five animals randomly selected per group were detected. Islets were identified using Image Pro Plus v.5.1 software (Media Cybernetics Inc.) for the calculation of islets area and total pancreas area. The pancreatic organ coefficient was the pancreas weight normalized by the body weight. The percentage of β-cell area was calculated as a ratio of β-cell area to the total pancreas area and multiplying this ratio by 100. The β-cell mass was calculated by multiplying the pancreas weight by the percentage of β-cell area as described [24], and normalized by the ratio of body weight between the tested animals and controls.

### Immunofluorescence Studies on Pancreatic Tissues

Detection of islet cells proliferation was performed by immunofluorescence assay. Tissues were processed as above and incubated with a mouse anti-proliferating cell nuclear antigen (PCNA) antibody (1∶2000 dilution; Sigma-Aldrich) overnight at 4°C. PCNA is shown present in the later G1, S, and G2 phases of the cell cycle, so it is a useful marker of cell proliferation [25]. Sections were then incubated by anti-mouse FITC secondary antibody (1∶500 dilution; Biyuntian Inc., China) for 2 h at room temperature. After that, sections were subjected to the immunofluorescence staining for insulin (1∶200 dilution; CST), followed by anti-rabbit CY3 secondary antibody (1∶500 dilution; Biyuntian Inc., China) for 2 h at room temperature. Nuclei were counterstained with 4, 6-diamidino-2-phenylindole (DAPI) (Sigma-Aldrich). Sections were imaged with an Olympus BX-61 microscope and analyzed with Image Pro Plus v.5.1 software (Media Cybernetics Inc.). Three islets per section (five sections per animal; five animals per group) were quantified for analysis. The percentage of PCNA^+^ β-cells was used to assess islet cells proliferation.

To evaluate islet apoptotic cells, sections were performed using a terminal deoxynucleotidyl transferase-mediated dUTP nick end labeling (TUNEL) assay (Roche) with insulin colocalization. Briefly, after deparaffinized in xylene and rehydrated in ethanol, tissues were subjected to antigen retrieval in 10 mmol/l citrate buffer (pH 3.0) and blocking with 10% (v/v) goat serum. Following the immunofluorescence staining for insulin, tissues were subjected to the TUNEL assay. After permeabilized in 0.3% (v/v) Triton X-100 for 8 min at 37°C, sections were then incubated with the FITC-conjugated TUNEL enzyme for 60°C min to detect DNA fragmentation. The same number of sections were quantified for apoptosis and reported as the percentage of TUNEL^+^ β-cells [25].

### Western Blot Analysis

Pancreatic islets from F1 mice were isolated by collagenase digestion. Islets were washed with PBS (phosphatebuffered saline) and lysed in RIPA lysis buffer (Sigma-Aldrich) with protease inhibitor. The protein lysates were sonicated and protein concentrations were determined by BCA kit (Thermo). For immunoblot analysis, total protein (40 µg) was separated by 10% SDS/PAGE and then transferred to a PVDF microporous membranes (Millipore). The membranes were blocked in 5% non-fat dried milk. Blots were then incubated with the following antibodies at 4°C overnight: anti-cyclin D1 (1∶1000 dilution; CST), anti-cleaved caspase 3 (1∶1000 dilution; CST), or anti-β-actin (1∶10000 dilution; Sigma-Aldrich). After three washings, membranes were incubated at room temperature with anti-mouse or anti-rabbit horseradish peroxidase-conjugated IgG (1∶50000 dilution; Jacksonimmuno). Immunoreactivity was detected using an enhanced chemiluminescence reaction (Millipore). Densitometric analysis employed Image J (NIH, USA).

### Statistics

Data were expressed as mean ± SE. The unpaired Student’s *t* test and repeated-measures one-way ANOVA were used as appropriate for comparison between groups of mice. These tests were performed with SPSS 13.0. The statistically significance of all tests was set at P<0.05.

Pregnancy outcomes were analyzed using Pearson’s chisquare test. The frequency of successful pregnancies carried to term in the control group was used to determine the number in the experimental groups. The pregnancy success rates of experimental groups were then used to calculate the test statistics, which were compared to a chi-square distribution with one degree of freedom.

## Results

### Pregnancy Outcomes and Birth Phenotypes

**Figure 1:**
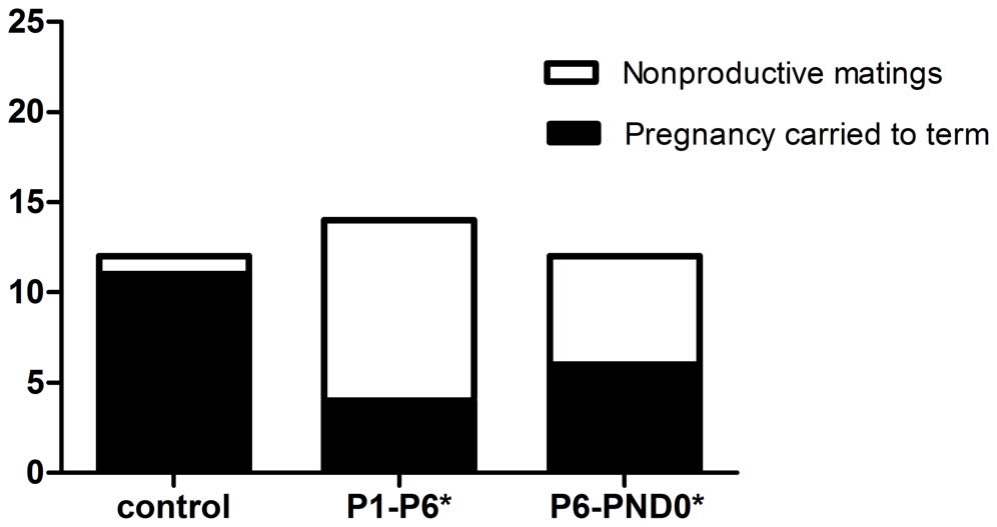
Effects of perinatal exposure to BPA on pregnancy outcomes. Exposure to BPA during preimplantation (P1–P6) or fetal period (P6-PND0) reduced the proportion of pregnancies carried to term. The number of copulations successfully carried to term in each of the groups was recorded via daily visual inspection. Data comparisons were made using Pearson’s chi-square test (*P1–P6: x^2^ = 10.54, P<0.01; *P6-PND0: x^2^ = 5.04, P<0.05).

In F0 female mice, reductions in pregnancies carried to term were observed in both P1–P6 and P6-PND0 groups, with a particularly significant decline in the former (P<0.05) (Fig. 1). The frequency of full-term pregnancies in the control group was over 90% (11/12), while the experimentally observed successful pregnancy rates were 29% (4/14) for P1–P6 group and 50% (6/12) for P6-PND0 group respectively.

**Figure 2:**
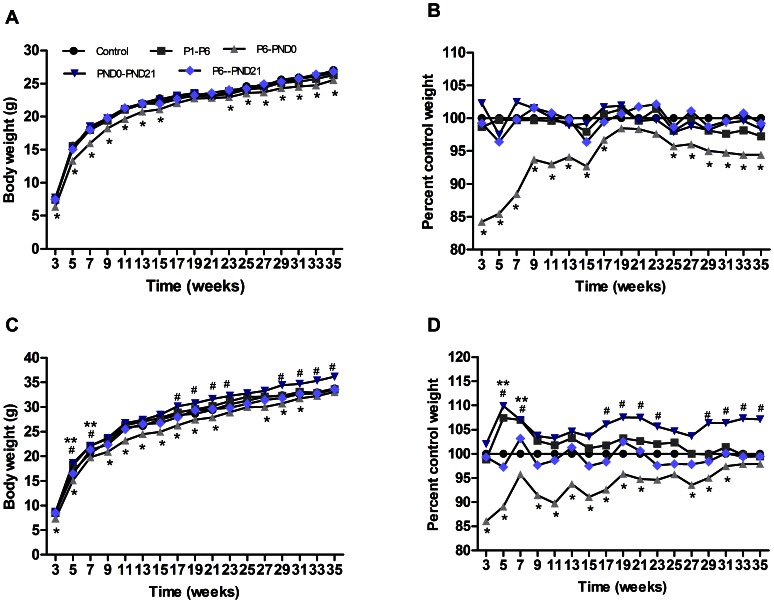
Postweaning body weight of offspring. Mean body weight (A) and percentage of control weight (B) from weaning to 35 weeks for females. Mean body weight (C) and percentage of control weight (D) from weaning to 35 weeks for males. *n* = 15–30 mice/group; some SEs are not visible for their low values. *p<0.05 for P6-PND0 mice compared with controls. ^#^p<0.05 for PND0-PND21 mice compared with controls. **p<0.05 for P1–P6 mice compared with controls.

In F1 offspring, BPA administration had no effect on birth sex, litter size, or birth weight in any treatment group (data not shown). However, both male and female pups of P6-PND0 group were lower in body weight than controls at weaning (P<0.05) (Fig. 2).

From 3 to 35 weeks of age, effects of maternal BPA exposure on postweaning body weight of offspring were sex and time-dependent. In female offspring, no difference on body weight were observed among P1–P6, PND0-PND21, P6-PND21 groups compared with control group; while P6-PND0 mice started gaining less weight than controls from weaning to 35 weeks (P<0.05) (Fig. 2A, B). In male offspring, body weight of P1–P6 mice was slightly higher than controls during 5 to 7 weeks of age (P<0.05) (Fig. 2C, D), and then comparable with controls from 9 weeks through about 8 months old. Interestingly, mice of neonatal exposure (PND0-PND21) gained more weight (P<0.05), while mice of fetal exposure (P6-PND0) gained less weight than controls (P<0.05), but for mice exposed to BPA during both fetal and neonatal time (P6-PND21), body weight were comparable with controls from weaning to 8 months of age (Fig. 2C, D).

### Glucose Homeostasis and Insulin Release

The effects of BPA administration on glucose homeostasis were also sex and time-dependent.

**Figure 3:**
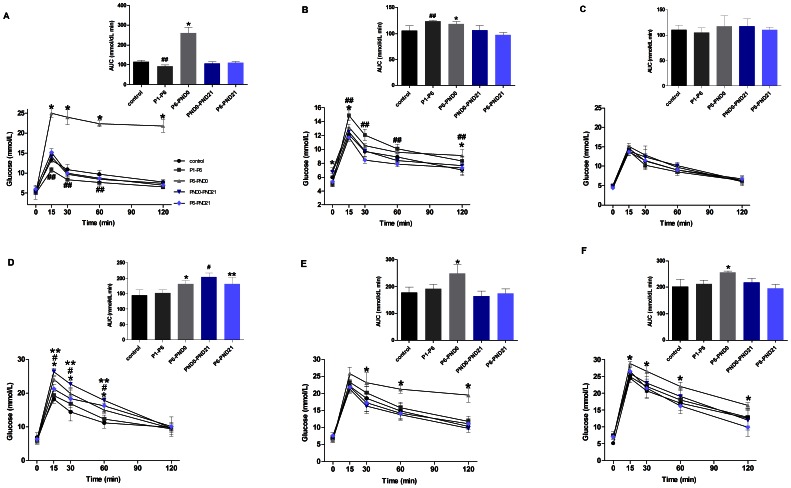
Blood glucose homeostasis in offspring shown by IPGTT. Serum glucose concentrations (mmol/L) following administration of glucose load (2 g/kg body weight) in fasted female (A, B and C) and male (D, E, and F) mice at 3 months (A, D), 6 months (B, E) and 8 months (C, F) of age. The mean total glucose AUC was shown by the insert at the same time point. Data are presented as mean ± SE (*n* = 10). Compared with controls, *p<0.05 for P6-PND0 mice; ^##^p<0.05 for P1–P6 mice; ^#^p<0.05 for PND0-PND21 mice; and **p<0.05 for P6-PND21 mice.

In female offspring, BPA exposure had no effect on glucose tolerance in PND0-PND21 and P6-PND21 groups from 3 months to 8 months (Fig. 3A, B, C). Following the glucose challenge, mice at 3 months of age in P6-PND0 group showed a higher total glucose response (AUC) to the glucose load relative to the controls (P<0.01). Furthermore, the peak glucose concentration at 15 min was significant higher (P<0.05) and the ability to clear the glucose load, determined by serum glucose concentrations at 120 min following the glucose challenge, was impaired (P<0.05) (Fig. 3A). The glucose intolerance in this group was also observed at 6 months old, with higher glucose concentrations at 120 min and AUC (P<0.05); meanwhile, the fasting glucose was also significant higher than controls (P<0.01) (Fig. 3B). However, in P1–P6 group, the glucose concentrations at 15 min, 30 min and 60 min, as well as AUC were lower than controls at 3 months old (P<0.05) (Fig. 3A); while at 6 months of age, both AUC and glucose concentrations were higher relative to controls, indicating the impaired glucose tolerance (P<0.05) (Fig. 3B). When at 8 months, no difference in glucose tolerance were observed among all groups (P>0.05) (Fig. 3C).

In male offspring, no difference in glucose tolerance was shown between P1–P6 and control group from 3 to 8 months (P>0.05) (Fig. 3D, E, F). At 3 months old, P6-PND0, PND0-PND21, as well as P6-PND21 mice showed a higher peak glucose concentration at 15 min than controls (P<0.05). Correspondingly, the AUC was significantly increased in these groups (P<0.05) (Fig. 3D). Besides, at the age of 6 months and 8 months, P6-PND0 mice remained an impaired glucose tolerance shown by the higher glucose concentration and AUC than controls (P<0.01); while in other groups, the glucose tolerance returned to normal (Fig. 3E, F).

Insulin release was also dectected in response to glucose load, and the insulinogenic index (ΔI_0–30_/ΔG_0–30_) was quantified to assess pancreatic β-cell secretory function in vivo. In female offspring, Δ_I0–30_/ΔG_0–30_ of P6-PND0 mice decreased significantly at 3 months old (P<0.05), but it improved dramatically nearly reaching the level of the controls (P = 0.7) at 6 months old, and retained normal at the age of 8 months. For P1–P6 mice, the insulin release was lower than that of controls just at 6 months old (P<0.01). No difference was found among PND0-PND21, P6-PND21 and control groups at any observed time point. Compared with the controls, the fasting insulin level of 3 m P6-PND0 mice was elevated, with the normal fasting glucose. And in 6 m mice, the fasting glucose was higher and the fasting insulin level was normal (Table 1).

**Table 1:** Effects of BPA on β-cell function in female offspring in vivo.

Age	Parameters	Control	P1–P6	P6-PND0	PND0-PND21	P6-PND21
3 months	Fasting glucose (mmol/L)	5.60±0.39	4.98±0.41	5.57±1.58	5.03±0.41	5.40±0.99
	Fasting insulin (µg/L)	0.46±0.01	0.52±0.09	0.53±0.01*	0.50±0.01	0.49±0.01
	ΔI30/ΔG30 (ng/mmol)	16.56±3.17	12.31±2.51	8.58±0.29*	15.06±3.02	13.15±1.85
6 months	Fasting glucose (mmol/L)	5.98±0.58	5.20±0.41	7.05±0.21*	6.36±0.59	5.34±0.63
	Fasting insulin (µg/L)	0.54±0.09	0.49±0.01	0.55±0.06	0.48±0.01	0.47±0.02
	ΔI30/ΔG30 (ng/mmol)	59.25±6.10	24.50±3.94*	47.65±5.21	50.55±5.11	51.25±4.92
8 months	Fasting glucose (mmol/L)	4.84±0.47	5.05±0.34	5.15±0.21	4.88±0.53	4.42±0.18
	Fasting insulin (µg/L)	0.57±0.08	0.55±0.04	0.51±0.06	0.55±0.03	0.57±0.02
	ΔI30/ΔG30 (ng/mmol)	24.09±3.17	31.48±2.58	32.97±1.64	23.81±3.63	22.21±2.95

Values are presented as mean ± SE. Values with an asterisk are significantly different from the controls (P<0.05).

In male offspring, ΔI_0−30_/ΔG_0−30_ of P6-PND0, PND0-PND21 and P6-PND21 mice were all reduced significantly at the age of 3 months (P<0.05). At 6 months of age, P6-PND0 group remained lower (P<0.01) while PND0-PND21 and P6-PND21 groups became higher (P<0.05). In 8-month-old mice, there was no abnormality observed in insulin releasing function among any BPA-exposed group. Distinct from P1–P6 female mice, the insulin response of P1–P6 male mice kept normal during the whole research process. Similar to female offspring, the higher fasting insulin level in the presence of normal fasting glucose were shown in P6-PND0, PND0-PND21 and P6-PND21 male mice suggesting a condition of insulin resistance (Table 2).

**Table 2:** Effects of BPA on β-cell function in male offspring in vivo.

Age	Parameters	Control	P1–P6	P6-PND0	PND0-PND21	P6-PND21
3 months	Fasting glucose (mmol/L)	5.77±1.06	5.43±0.90	5.63±0.56	5.33±1.02	5.53±0.87
	Fasting insulin (µg/L)	0.51±0.03	0.53±0.01	0.57±0.01*	0.57±0.02*	0.55±0.01*
	ΔI30/ΔG30 (ng/mmol)	16.19±1.68	16.36±1.53	9.57±2.10*	10.42±1.95*	10.38±1.89*
6 months	Fasting glucose (mmol/L)	6. 67±0.49	6.8±0.35	6.25±0.76	7.08±0.34	7.64±0.86
	Fasting insulin (µg/L)	0.65±0.09	0.94±0.01	1.22±0.06*	0.73±0.01	0.75±0.02
	ΔI30/ΔG30 (ng/mmol)	55.86±6.14	54.72±5.99	25.1±2.37*	84.28±5.73*	120.53±5.50*
8 months	Fasting glucose (mmol/L)	5.15±0.35	7.50±1.21	6.88±0.34	7.03±0.64	7.14±0.76
	Fasting insulin (µg/L)	0.53±0.03	0.58±0.04	0.59±0.01*	0.58±0.01	0.52±0.00
	ΔI30/ΔG30 (ng/mmol)	73.43±4.67	73.90±6.70	64.00±4.12	66.40±5.23	60.25±5.11

Values are presented as mean ± SE. Values with an asterisk are significantly different from the controls (P<0.05).

### Assessment of Insulin Sensitivity

**Figure 4:**
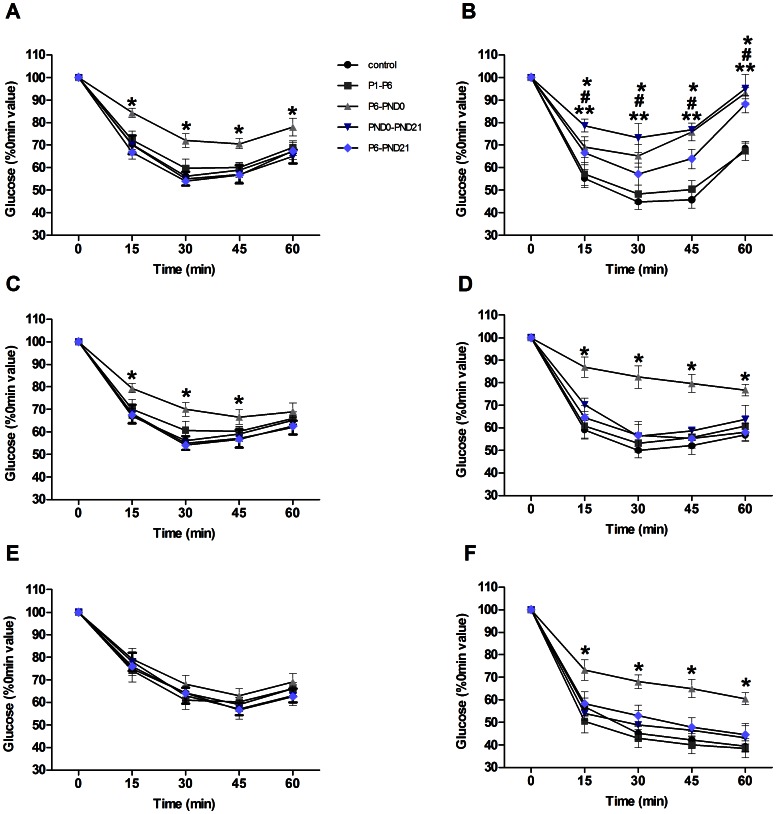
Insulin sensitivity in offspring shown by IPITT. Blood glucose levels before and after i.p. injection of insulin (1 IU/kg body weight) in fed female (A, C and E) and male (B, D, and F) mice at 3 months (A, B), 6 months (C, D) and 8 months (E, F) of age. Blood glucose levels were normalized to those at 0 min (100%). Data are presented as mean ± SE (*n* = 10). Compared with controls, *p<0.05 for P6-PND0 mice; ^#^p<0.05 for PND0-PND21 mice; and **p<0.05 for P6-PND21 mice.

The IPITT was performed to further evaluate insulin sensitivity. In female offspring, response during ITT was unaltered in P1–P6, PND0-PND21, P6-PND21 and control groups (Fig. 4A, C, E). At 3 and 6 months, blood glucose decrease were less pronounced in P6-PND0 mice after administration of insulin than that in controls (P<0.05), confirming a reduction of insulin sensitivity (Fig. 4A, C). In male, the P6-PND0, PND0-PND21 and P6-PND21 mice at the age of 3 months showed an attenuated response to insulin, with the glucose levels 15 min, 30 min, 45 min and 60 min after the insulin injection being significantly higher than in controls (P<0.05) (Fig. 4B). And the impaired insulin sensitivity was persisted in P6-PND0 up to 8 months (P<0.05) (Fig. 4B, D, F).

### Insulin Secretion after Glucose Stimulation in vitro

**Figure 5:**
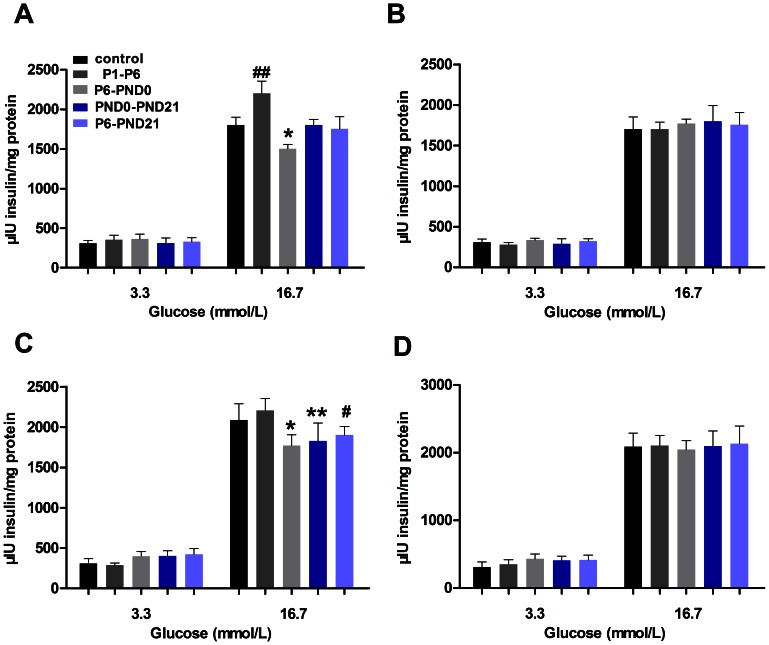
Glucose-stimulated insulin secretion from isolated islets in offspring. Islets from female (A, B) and male (C, D) offspring were incubated with the indicated glucose concentrations for 1 h, and the accumulated amount of insulin was determined. A and C, at 3 months old; B and D, at 8 months old. Results are expressed as as mean ± SE of eight replicates from three independent isolations. Compared with controls, ^##^p<0.05 for P1–P6 mice; *p<0.05 for P6-PND0 mice; **p<0.05 for PND0-PND21 mice; and ^#^p<0.05 for P6-PND21 mice.

In order to eliminate the interferences of insulin resistance and other influencing factors, we also conducted GSIS test of islet in vitro to evaluate β-cell function. BPA exposure did not significantly affect basal insulin release. Compared with controls, islets isolated from BPA-exposed offspring tended to release more insulin in response to 3.3 mM glucose stimulus, but the difference was not significant (P>0.05). At 3 months old, islets from P1–P6 female mice displayed an elevated secretion evoked by 16.7 mM glucose (P<0.05) (Fig. 5A). In contrast to the increased GSIS, islets from female in P6-PND0 and male in P6-PND0, PND0-PND21 and P6-PND21 groups, all exhibited a significantly inhibition of the secretory response to 16.7 mM glucose (P<0.05) (Fig. 5A, C). When at 8 months old, no apparent difference was displayed in insulin secretion among the groups (Fig. 5B, D). The β-cell secretory function assayed in vitro was consistent with the insulin release in vivo.

### Islet Morphometric Analysis

**Figure 6:**
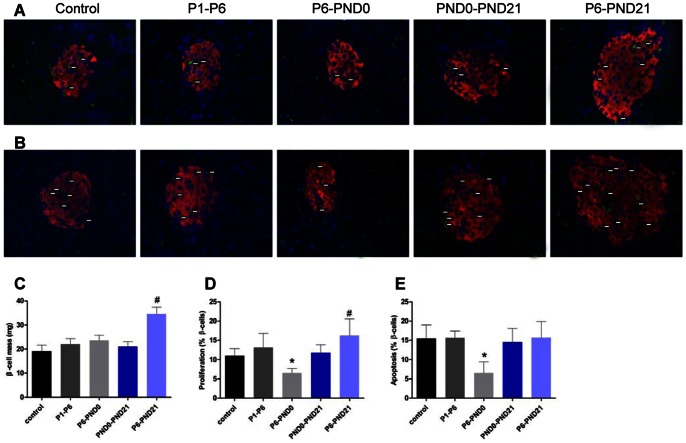
β-cell morphological analysis in female offspring at 3 months of age. A, PCNA^+^ islet cells were revealed by indirect immunofluorescence using anti-PCNA (green) and antiinsulin (red) antibodies, and nuclei were counterstained with DAPI (blue) (X 400).White arrows marked PCNA^+^ cells. B, Representative pancreatic sections immunostained with TUNEL (green) and antiinsulin (red) antibodies, and nuclei were counterstained with DAPI (blue). White arrows marked TUNEL^+^ cells. C, β-cell mass. The PCNA^+^ islet cells (D) and TUNEL^+^ islet cells (E) were quantified. Data are presented as mean ± SE of five sections from five independent isolations. *p<0.05 for P6-PND0 mice compared with controls. ^#^p<0.05 for P6-PND21 mice compared with controls.

**Figure 7:**
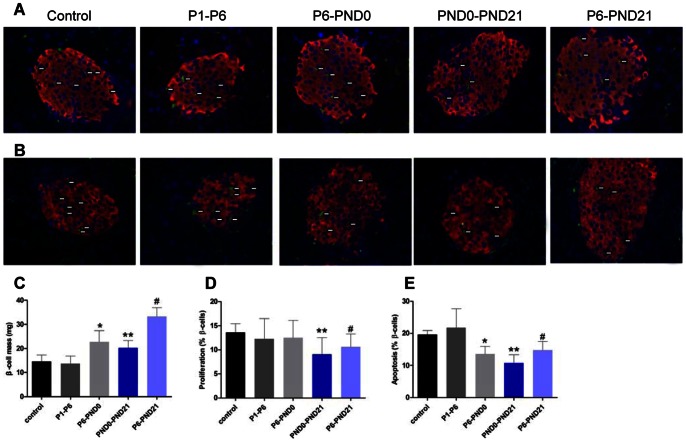
β-cell morphological analysis in male offspring at 3 months of age. A, PCNA^+^ islet cells were revealed by indirect immunofluorescence using anti-PCNA (green) and antiinsulin (red) antibodies, and nuclei were counterstained with DAPI (blue) (X 400).White arrows marked PCNA^+^ cells. B, Representative pancreatic sections immunostained with TUNEL (green) and antiinsulin (red) antibodies, and nuclei were counterstained with DAPI (blue). White arrows marked TUNEL^+^ cells. C, β-cell mass. The PCNA^+^ islet cells (D) and TUNEL^+^ islet cells (E) were quantified. Data are presented as mean ± SE of five sections from five independent isolations. Compared with controls, *p<0.05 for P6-PND0 mice; ^#^p<0.05 for P6-PND21 mice; and **p<0.05 for PND0-PND21 mice.

We next studied whether pancreatic β-cell mass was altered in BPA-treated mice. At 3 and 8 months of age, no changes were shown in pancreatic organ coefficient among the groups (Table 3, 4). In female offspring, pancreatic β-cell mass was significantly increased in P6-PND21 mice (P<0.01), while other groups were comparable with controls (Figure 6C). In male, the β-cell mass of BPA-treated groups (P6-PND0, PND0-PND21 and P6-PND21) were all higher relative to controls (P<0.05) (Figure 7C). However, BPA exposure during P1–P6 showed no effect on the β-cell mass regardless of sex (Figure 6C, 7C).

**Table 3:** β-cell morphological analysis in female offspring.

Age	Parameters	control	P1–P6	P6-PND0	PND0-PND21	P6-PND21
3 months	pancreatic organ coefficient (%)	1.36±0.06	1.43±0.10	1.42±0.08	1.37±0.13	1.33±0.12
8 months	pancreatic organ coefficient (%)	1.16±0.13	1.21±0.15	1.18±0.02	1.12±0.14	1.06±0.10
	β-cell mass (mg)	26.38±4.02	28.39±6.68	22.41±3.62	23.52±5.02	22.93±3.93
	proliferation (% β-cells)	10.35±1.37	11.30±1.48	9.33±1.97	9.03±1.46	8.76±1.45
	apoptosis (% β-cells)	7.61±1.68	10.41±2.30	8.07±1.52	9.26±2.92	7.89±2.30

Values are presented as mean ± SE.

**Table 4:** β-cell morphological analysis in male offspring.

Age	Parameters	control	P1–P6	P6-PND0	PND0-PND21	P6-PND21
3 months	pancreatic organ coefficient (%)	1.43±0.12	1.25±0.19	1.46±0.13	1.24±0.20	1.44±0.07
8 months	pancreatic organ coefficient (%)	0.92±0.07	0.95±0.13	0.87±0.13	0.87±0.11	0.86±0.07
	β-cell mass (mg)	29.30±6.25	23.29±4.76	24.55±4.62	25.93±5.40	31.21±6.81
	proliferation (% β-cells)	11.98±1.71	11.73±2.40	11.08±2.67	10.09±2.10	11.66±3.02
	apoptosis (% β-cells)	11.55±2.65	10.43±1.43	11.52±2.21	11.61±2.34	11.19±2.89

Values are presented as mean ± SE.

To determine whether the change in β-cell mass was associated with β-cell proliferation, apoptosis or neogenesis, we detected the percentage of PCNA^+^ islet cells and TUNEL^+^ islet cells. At the age of 3 months, the percentage of PCNA^+^ islet cells was markedly reduced in P6-PND0 female (P<0.05) (Fig. 6A, D), PND0-PND21 male and P6-PND21 male mice (P<0.05) (Fig. 7A, D), accompanied by the reduced percentage of TUNEL^+^ islet cells relative to controls (P<0.05) (Fig. 6B, 6E, 7B and 7E). These changes indicated a decreased rate of β-cell turnover. In addition, the proliferating islet cells increased in P6-PND21 female mice (P<0.05) (Fig. 6A, D), and apoptotic islet cells decreased in P6-PND0 male mice (P<0.05) (Fig. 7B, E). This suggested that the change of β-cell mass was partly related to proliferation and apoptosis, but the role of neogenesis could not be excluded. In deed, there were newly formed ductassociated islet cells observed in each group, especially evident in BPA-treated groups P6-PND0, PND0-PND21 and P6-PND21 (data not shown).

At 8 months, for both sexes, the relationship between maternal BPA treatment and β-cell mass, as well as β-cell turnover was lost. There was no difference on these parameters of BPA-treated groups compared with controls (Table 3, 4).

**Figure 8:**
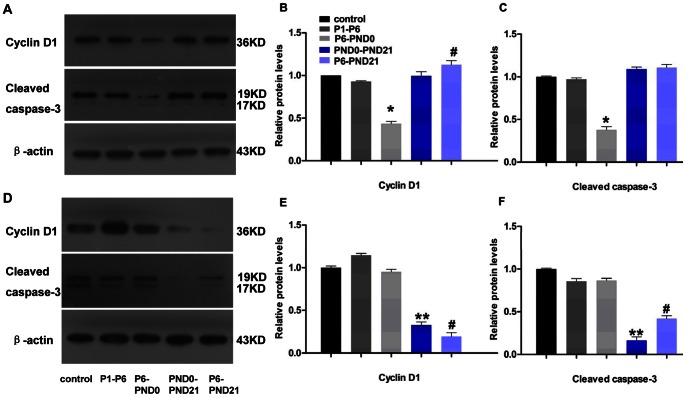
Western blot analysis of cyclin D1 and cleaved caspase-3 in offspring at 3 months of age. A, B and C, female; and D, E and F, male. A total of 40 µg of protein lysate from isolated mice islets was run with β-actin used as a loading control. Blot is representative of three independent experiments from three different organ donors. B, C, D and E: The density of expression levels was quantified after scanning, normalized to actin levels, and expressed as change from control, respectively. Data are presented as mean ± SE. Compared with controls, *p<0.05 for P6-PND0 mice; ^#^p<0.05 for P6-PND21 mice; and **p<0.05 for PND0-PND21 mice.

To further validate these results, we measured the cleaved caspase-3 protein level to assess apoptosis and cyclin D1 for proliferation by western blot analysis. Cleaved caspase-3 represents a measure of apoptosis in cells with an intact plasma membrane. And cyclin D1 is a major cell-cycle regulator involved in progressing cells to the proliferative stage. The effect of BPA exposure in diminishing the rate of β-cell turnover was further confirmed, with the expression levels of cleaved caspase-3 and cyclin D1 were lower in P6-PND0 female, PND0-PND21 male and P6-PND21 male mice (Fig. 8).

## Discussion

The well-known “fetal plasticity” theory is that conditions experienced in utero can have lifelong effects on health [26]. In the present study, we demonstrated that exposure to low dose of BPA during critical periods of development resulted in endocrine and metabolic changes, especially an adverse effect on adult glucose homeostasis.

Data from present study revealed time and sex-dependent effects of maternal BPA exposure on body weight in offspring. The body weight of postweaning mice fetally exposed to BPA was lower, while neonatal males’ of BPA exposure remained heavier than controls. However, combination of fetal and neonatal BPA exposure did not alter the growth rate of offspring, whose body weight remained comparable with controls. These observations are partly in accordance with some previous publications, such as reduced postnatal growth after fetal exposure [14], the increased body weight of neonatal treatment [5], and no difference when exposed from fetal period to weaning [27], [28]. However, some distinct works report no alterations or converse changes of body weight in pups exposed to BPA perinatally [15], [16], [29]. The cause of such discrepancies is unclear, but it might be related to dose, time, sex and species. In addition, it calls attention to methodological and statistical concerns, such as: 1) the way of BPA administration; 2) unpurified chow diets containing phytoestrogens; and 3) inadequate sample size [30]. The present data showed the difference in alteration of body weight after BPA exposure during different developmental periods. One possible explanation is that white adipose tissue formation begins before birth, but its differentiation or expansion mainly takes place in postnatal life [31]. In addition, the effect of BPA on body weight maybe due to its regulation on postnatal food intake and metabolism change [17].

As we know, drastic change of body weight may pose a great risk to type 2 diabetes. So we next investigated the glucose metabolism. Indeed, our results demonstrated that BPA exposure during prenatal and/or postnatal period could impair adult glucose homeostasis. Compared with control group, not only P6-PND0 female and male offspring weighted lighter, but also heavyer PND0-PND21 male mice all showed glucose intolerance. It is likely that early BPA exposure can influence several mechanisms important for body weight regulation, including adipocyte deposition, glucose uptake and homeostasis, as well as the development and maturation pathways closely associated with energy homeostasis [17]. However, blood glucose regulation is not always relevant to body changes. Our study showed that P6-PND21 male mice, whose body weight was comparable with those of controls, developed glucose intolerance at the age of 3 months. Several studies also report the effect of BPA exposure in elevating body weight but not impairing glucose tolerance [12]. In addition, the present study demonstrated that the effects of BPA exposure on glucose homeostasis were sex-dependent, and male was more susceptible to damage of BPA. The sex difference maybe in part due to the protective effect of physiological estrogens in females against diabetes [5], [32].

The pathogenesis of type 2 diabetes is complex. In most instances, it develops due to a progressive reduction in the response of the pancreas to produce sufficient insulin to compensate for insulin resistance [33], [34]. That means it clearly requires defects in both β-cell function and insulin sensitivity.

Interestingly, the higher fasting insulin level in BPA-treated mice in the presence of normal glucose suggests a condition of insulin resistance. Moreover, insulin tolerance was also significantly impaired in males, further confirming reduced insulin sensitivity. Therefore, perinatally BPA-exposed mice, especially males, showed a complex alteration of glucose homeostasis.

To further investigate the role of β-cell dysfunction in BPA-induced impairment of glucose homeostasis, we assessed the β-cell function of insulin secretion both in vivo and in vitro. We noticed that both insulin secretion and sensitivity were reduced in BPA-treated mice. It is well known that relationship between insulin sensitivity and insulin secretion is not linear but hyperbolic under normal conditions [35]. In the present study, the inability of insulin secretion to compensate for a decrease in insulin sensitivity resulted in glucose intolerance.

The mechanism underlying β-cell dysfunction has not been elucidated, but two hypotheses are possible, the partial loss of mass or purely functional defect of insulin secretion [36]. In this study, our data suggested that the β-cell dysfunction was largely a functional issue, rather than a morphological abnormality. There was no reduction in β-cell mass in any BPA-treated group. Conversely, the β-cell mass remained invariable or even increased, without concomitant improvement in insulin secretion. Islet β-cell mass is mainly coordinately regulated by changes in cell replication, neogenesis, and apoptosis [24]. So we next determined whether the increase in β-cell mass was associated with increased replication, decreased apoptosis or both. Except the female P6-PND21 mice showed increased cell proliferation and invariable apoptosis, β cells proliferation reduced or remained constant in other groups, with the same trend of changes in apoptotic cells. And these results were further validated by western blot analysis for the protein expression levels of cyclin D1 and cleaved caspase-3. So the increase of β-cell mass maybe due to the attenuating β-cell turnover, or the enhanced neogenesis of β cells from ducts. However, quantitation of neogenesis is difficult to carry out, for lacking of markers for new β cells or time denominators for the process [37]. The definition of neogenesis used in this study includes β cells budding from the ducts or scattered cells in the exocrine pancreas [24]. Based on this standard, numerous islets budding from ductal epithelium were shown in BPA-treated mice, although this was not quantified.

In addition, we found that functional changes were not consistent with morphological changes considering the compensation function of β cells. In general, β cells can increase insulin secretion sufficiently to overcome insulin resistance and maintain euglycaemia. However, after exposure to BPA, the β-cell mass increased while insulin secretion reduced or remained invariable, which suggested that β cells were less functional in BPA-exposed mice. These less functional cells maybe resulted from the neogenesis of β cells with incomplete function or low turnover rate making the cells aging or exhausted. These observations are consistent with the study of dietary-fat-induced obesity in mice [38] and lipid infusion in rats [39] etc, which represent a disassociation between β-cell mass and secretory function. These results confirmed that adult β-cell dysfunction induced by maternal exposure to BPA was mainly a functional abnormality.

Moreover, our findings showed that the effects of perinatal exposure to BPA on adult glucose homeostasis were strongly stage-dependent. The fetal and neonatal development periods especially fetal stage may be the critical window of susceptibility to BPA exposure. But the mechanism remained to be determined. It is perhaps related to some factors: First, different major periods exist in pancreatic ontogeny in rodent, at least two critical developmental windows: a primary transition from embryonic day (E) 9.5 to birth and the postnatal period from birth to weaning. The former covers the initial embryogenic process of endocrine cell formation; the later appears with β-cell proliferation, functional maturation and establishment of β-cell mass [40], [41]. These developmental changes make the animals suited to metabolic regulation in later adult life. So BPA exposure during different periods may result in different changes of pancreas development, such as the timing or amplitude of development, the quantity and quality of β-cell population and ultimately leading to glucose intolerance. It is likely that increase of β-cell mass occurs during critical developmental windows such as neonatal stage. Second, previous researches indicate the effects of BPA are a non-monotonic dose–response [5], [17], which makes it possible for low doses of BPA to be more effective than high doses in altering metabolic homeostasis. In our study, the accumulation of BPA in offspring may be different among groups with different continuing exposure time. It is possible that shorter exposure time induced more insults than longer time, just as P6-PND0 group compared with P6-PNDD21. Third, recent reports show that BPA may be stored in adipose tissue [19], [42], [43], [44]. This provides the chance for stored BPA in body to affect the adult metabolism.

In conclusion, our results point out that BPA exposure during pregnancy could induce the long-term changes in adult glucose homeostasis in the offspring. Both fetal and neonatal periods are susceptible to BPA exposure, especially for fetal stage in which the β-cell dysfunction persists for a long time till the observed end point. However, additional studies still need to be done to investigate the effects and mechanism of BPA exposure on pancreatic development. In applying these results to the human population, the developmental differences between species should be considered. In rodent, pancreatic development occurs in both fetal and neonatal stages, whereas in humans, the major development is completed prenatally [25]. In any case, the impairment of early pancreatic development induced by BPA exposure will result in long-term changes. And BPA exposure should be considered as a possible risk factor for type 2 diabetes.
